# Supplementary material for: Characterization of Composition and Antifungal Properties of Leaf Secondary Metabolites from Thirteen Cultivars of Chrysanthemum morifolium Ramat
Source: Molecules. 2019 Nov 20;24(23):4202. doi: 10.3390/molecules24234202 (PMC6935761; doi:10.3390/molecules24234202)
Supplement: Supplementary file 1 [file molecules-24-04202-s001.pdf]

Supplementary materials:

Table S1. Apolar secondary metabolites identified in the leaf extracts of 13 cultivars of *C. morifolium* (μg g^{-1})

NO.	Compounds	Retention Index[a]	Cultivars												
			**Cm1**	**Cm2**	**Cm3**	**Cm4**	**Cm5**	**Cm6**	**Cm7**	**Cm8**	**Cm9**	**Cm10**	**Cm11**	**Cm12**	**Cm13**
	**Terpenoids**														
	**Monoterpenoids**														
1	fenchone	938	1.95 ±0.54[a]	2.05 ±1.00	5.76 ±2.97	1.98 ±0.03	ND	ND	5.73 ±3.58	ND	1.30 ±0.24	2.50 ±0.58	4.32 ±0.50	1.71 ±0.38	2.46 ±0.34
2	limonene	1033	ND[b]	ND	ND	ND	ND	ND	5.57 ±2.73	1.02 ±0.26	ND	ND	8.02 ±1.11	0.67 ±0.05	ND
3	1,8-cineole	1037	19.98 ±1.93	31.29 ±19.33	8.91 ±3.90	1.25 ±0.10	14.65 ±1.77	2.64 ±1.40	6.47 ±2.09	4.28 ±0.82	20.59 ±2.59	28.23 ±5.59	19.05 ±1.34	19.77 ±3.87	36.35 ±4.60
4	filifolone	1110	ND	ND	8.40 ±1.69 [b]	4.64 ±0.47	ND	ND	ND	ND	ND	ND	ND	4.92 ±2.35	16.53 ±1.95
5	allo-ocimene	1134	ND	ND	4.65 ±2.60	2.92 ±0.41	20.23 ±1.41	ND	1.62 ±0.38	ND	12.59 ±0.79	2.66 ±0.63	3.24 ±1.54	16.89 ±0.81	ND
6	camphor	1151	33.95 ±3.10	ND	2.90 ±0.89	ND	3.32 ±0.41	ND	17.51 ±4.73	1.69 ±0.41	44.51 ±3.48	8.20 ±1.46	11.48 ±1.15	4.69 ±0.14	3.79 ±0.59

7	endo-borneol	1172	51.03 ±4.50	16.99 ±8.91	10.27 ±1.40	ND	12.56 ±1.04	8.17 ±3.85	6.52 ±3.16	2.86 ±0.22	7.93 ±0.80	ND	50.51 ±4.33	19.13 ±1.99	ND
8	(*E*)-ocimenone	1247	ND	ND	4.52 ±0.84	2.47 ±0.32	ND	ND	ND	ND	ND	3.31 ±0.82	ND	ND	10.34 ±1.06
9	isopiperitenone	1279	ND	ND	9.01 ±1.75	ND	ND	ND	ND	ND	ND	6.77 ±1.91	ND	ND	19.02 ±1.95
	**Sesquiterpenoids**														
10	β-elemene	1401	ND	ND	18.12 ±4.74	9.49 ±2.02	ND	20.88 ±3.11	20.27 ±4.73	25.96 ±4.91	84.42 ±5.30	12.79 ±4.84	33.21 ±3.58	11.50 ±6.29	39.65 ±3.82
11	β-ylangene	1430	30.72 ±7.50	39.48 ±20.49	41.42 ±5.13	62.83 ±6.36	141.42 ±5.38	29.00 ±5.37	32.04 ±7.65	24.92 ±5.04	95.04 ±5.99	47.85 ±14.53	95.72 ±27.17	131.12 ±7.22	35.64 ±7.15
12	epi-bicyclosesquiphellandrene	1440	8.98 ±2.13	12.49 ±6.90	11.91 ±3.53	11.64 ±1.72	31.42 ±2.15	7.71 ±1.47	8.70 ±2.13	9.30 ±2.33	18.75 ±1.88	13.13 ±3.69	36.53 ±12.79	30.51 ±1.19	14.91 ±2.69
13	γ-muurolene	1455	3.50 ±0.23	ND	4.57 ±1.60	4.64 ±0.58	11.70 ±0.79	2.70 ±0.48	3.00 ±0.76	3.36 ±0.96	6.88 ±0.70	4.94 ±1.53	13.80 ±5.01	11.23 ±0.39	5.47 ±0.91
14	(*E*)-β-farnesene	1464	8.14 ±1.89	10.49 ±6.70	9.87 ±2.08	19.75 ±2.93	27.80 ±1.41	6.06 ±1.36	7.06 ±1.69	8.42 ±1.68	29.65 ±2.17	14.91 ±3.96	25.27 ±7.95	25.75 ±0.76	7.09 ±4.91
15	germacra-1(10),4(15),5-triene	1488	21.55 ±3.62	ND	7.41 ±1.59	35.15 ±11.69	8.01 ±3.04	ND	ND	18.03 ±12.84	ND	10.24 ±7.87	7.33 ±4.08	7.37 ±0.03	ND
16	α-curcumene	1491	32.05 ±5.69	37.49 ±19.03	49.78 ±9.06	64.97 ±2.15	148.68 ±8.55	23.01 ±2.88	24.72 ±4.88	37.43 ±4.95	84.83 ±54.05	79.32 ±31.77	126.57 ±24.09	156.06 ±23.80	43.16 ±9.63
17	β-selinene	1494	4.53 ±1.09	ND	ND	15.04 ±2.65	ND	ND	ND	ND	13.06 ±0.06	93.93 ±26.90	ND	ND	66.15 ±9.66
18	zingiberene	1503	ND	ND	111.26 ±7.00	184.02 ±35.24	116.33 ±22.98	ND	7.99 ±2.47	46.30 ±19.02	204.17 ±96.78	164.07 ±92.97	105.09 ±32.97	94.61 ±29.92	ND

19	γ-cadinene	1508	1.91 ±0.83	5.79 ±2.85	7.29 ±1.85	ND	13.13 ±2.35	ND	ND	5.68 ±2.06	10.49 ±1.88	6.78 ±2.71	17.08 ±5.62	13.60 ±0.96	6.02 ±1.55
20	α-farnesene	1515	ND	29.66 ±20.44	22.72 ±5.45	38.25 ±3.37	51.08 ±2.90	14.18 ±2.48	8.46 ±2.97	11.68 ±0.66	10.55 ±0.59	42.40 ±13.01	21.56 ±2.30	46.02 ±3.13	9.25 ±1.91
21	β-sesquiphellandrene	1532	47.25 ±12.11	4.46 ±3.07	5.86 ±1.00	119.43 ±13.50	24.08 ±0.57	10.37 ±0.77	ND	29.16 ±3.41	129.61 ±5.68	18.97 ±4.90	9.90 ±1.18	20.93 ±1.67	ND
22	germacrene D-4-ol	1587	ND	9.70 ±5.11	9.16 ±3.54	ND	27.64 ±1.72	ND	ND	ND	16.81 ±3.76	9.64 ±2.63	11.51 ±5.26	24.36 ±1.44	12.20 ±1.57
23	α-patchoulene	1605	ND	24.39 ±11.73	ND	ND	ND	18.89 ±2.51	18.23 ±4.56	ND	28.52 ±1.69	12.21 ±2.98	ND	ND	ND
24	selina-6-en-4-ol	1668	15.99 ±3.82	20.57 ±13.16	ND	ND	ND	ND	ND	ND	ND	ND	ND	ND	ND
25	longifolenaldehyde	1745	8.41 ±0.96	ND	ND	ND	ND	ND	ND	ND	ND	ND	ND	ND	ND
	**Diterpenoids**														
26	phytol	2120	23.85 ±2.81	14.69 ±14.34	18.16 ±8.70	16.53 ±3.70	7.35 ±1.22	23.55 ±13.78	27.08 ±10.27	17.65 ±2.85	9.32 ±3.54	ND	ND	5.58 ±3.85	45.81 ±27.39
	**Fatty acid derivatives**														
27	1-octanol, 2-butyl-	1085	ND	16.85 ±12.35	ND	ND	ND	ND	ND	1.82 ±0.26	ND	ND	ND	ND	ND
28	tetradecane, 2,6,10-trimethyl-	1497	43.74 ±3.11	ND	ND	ND	ND	ND	ND	ND	ND	ND	ND	ND	ND
29	cryptomeridiol	1826	7.59 ±2.12	ND	ND	ND	ND	ND	1.98 ±1.22	ND	ND	10.89 ±1.60	ND	ND	ND

30	8,9-epoxyacorenon-B	1833	ND	ND	ND	3.94 ±3.39	10.93 ±7.80	ND	ND	ND	18.71 ±18.90	19.28 ±16.29	ND	17.02 ±5.95	ND
31	neophytadiene	1846	14.61 ±10.15	46.66 ±58.10	27.54 ±11.58	45.63 ±10.21	27.52 ±14.04	25.88 ±3.83	24.83 ±2.84	27.22 ±4.44	15.61 ±1.90	7.52 ±2.29	9.65 ±7.75	10.56 ±4.27	12.72 ±3.99
32	phytyl acetate	1870	ND	6.84 ±8.20	4.20 ±1.53	6.84 ±1.93	5.13 ±2.07	5.11 ±3.43	3.34 ±0.53	6.06 ±0.99	ND	2.88 ±1.77	ND	3.33 ±1.78	ND
33	3,7,11,15-tetramethyl-2-hexadecen-1-ol	1888	5.05 ±3.77	15.73 ±19.00	8.55 ±6.03	11.82 ±2.99	7.62 ±5.75	8.78 ±0.55	8.43 ±1.31	10.41 ±1.61	4.48 ±0.66	ND	ND	3.25 ±2.22	ND
34	(Z)-2-(hexa-2,4-diyn-1-ylidene)-1,6-dioxaspiro[4.4]non-3-ene	1892	7.84 ±4.95	ND	ND	180.63 ±0.25	ND	ND	ND	ND	63.76 ±2.58	69.69 ±23.78	ND	ND	ND
35	spiroether (E)	1905	ND	2.15 ±1.59	ND	113.00 ±14.44	ND	ND	ND	ND	15.52 ±10.46	77.19 ±10.15	ND	ND	1.49 ±0.12
	**Benzenoids**														
36	3,5-di-tert-butylphenol	1519	ND	53.35 ±39.73	ND	ND	ND	ND	ND	ND	ND	ND	ND	ND	ND
37	1-isopropenylnaphthalene	2283	ND	ND	ND	25.48 ±3.86	ND	ND	ND	ND	ND	ND	ND	ND	ND

[a] Mean SD, the mean values received by three samples from the same origin.

[b] Not detected.
